# A Case of Epilepsy Successfully Treated

**Published:** 1781-06

**Authors:** 


II. *A caſe of Epilepſy ſucceſsfully treated. By Mr.* Thomas Clark, *Surgeon at Market Harborough. Read* June 4, 1781.

IN the courſe of my practice, I frequently meet with patients who date the origin of their complaints from inoculation for the ſmall-pox. They will ſay, that they enjoyed a good ſtate of health from their infancy till they underwent inoculation, but that ſince that period they have been ſubject to ophthalmia, epilepſy, wandering pains in the limbs, impoſthumations, pofthumations, &c. Thefe complaints might perhaps have appeared after a recovery from the fmall-pox in the natural way, and poffibly from various other caufes; but at any rate it feems deferving the attention of medical practitioners, to determine by accurate and repeated obfervations whether thefe fufpicions are well-founded; and if the evil really exifts, to endeavour to obviate it by fuitable remedies.

In the cafes of this kind that have fallen under my own care, I have generally experienced good effects from mercurial purges, and antimonial emetics occafionally repeated; but I have never found any thing fo beneficial as an artificial difcharge by iffue or feton.—Of feveral inftances of the efficacy of the latter in this way, I fhall content myfelf with mentioning the following. If the Society fhould think it deferving of a place in their Journal, it will afford me great fatisfaction.

A child, at the age of three years, was inoculated for the fmall-pox, had a favourable eruption, and paffed through the difeafe without any unufual fymptoms. Six weeks after her recovery, an inflammatory tumour appeared in the axilla, which fuppurated and healed without difficulty. A few weeks after this fhe had a flight

ſlight attack of epilepſy, which for ſix weeks returned every ten or twelve days. The fits then occurred every five or ſix days, and afterwards became more and more frequent, till at length ſhe was attacked with them twice and ſometimes thrice every day. She appeared to be ſuddenly ſeized with pain, by her lifting her hand to her head, and would immediately fall down ſenſeleſs. In the ſpace of a minute ſhe would ſeem perfectly recovered, and play about the houſe as uſual. She had a knowledge of their approach, by her endeavouring to get to a chair when ſhe was going to fall, and appeared much frightened. Although the frequency of attack was ſo much increaſed, the duration of the fits was not longer than at their firſt appearance. Half a grain of *vitriol. alb.* as a ſubſtitute for the *flor. zinci*, was given twice a day for a fortnight; but the fits increaſing, I ventured to try the effects of a ſeton in the back, which I preferred to the neck, merely to avoid disfiguring her by the ſcar. I determined at the ſame time to give no medicine of any kind, that a perfect trial might be given to the diſcharge alone. The good effect of this drain ſoon became evident, by a gradual decreaſe of the attacks. A month after the introduction of the ſeton,

ſhe

ſhe remained ſeven days free from an attack, and in another month the fits entirely left her. The ſeton was then ſuffered to dry up, and the child appeared in good health in the day time, but was often feveriſh and thirſty in the night. I therefore directed emetic tartar to be given once a week in the morning faſting, in a doſe ſufficient to puke. This method, with attention to her diet, ſoon removed every appearance of fever, and the child remains perfectly well, it being now four years ſince ſhe was inoculated.

